# Exploring of the impact of AI feedback on college students' critical thinking: a intervention experiment based on design discipline

**DOI:** 10.3389/fpsyg.2026.1806913

**Published:** 2026-03-16

**Authors:** Jinhua Yang, Tianyue Niu

**Affiliations:** 1 The School of Humanities, Tongji University, Shanghai, China; 2 Xiamen Academy of Arts and Design, Fuzhou University, Xiamen, China

**Keywords:** artificial intelligence, critical thinking, design learning, feedback, mixed-methods study

## Abstract

**Introduction:**

Artificial intelligence-driven feedback and assessment have become increasingly important teaching approaches in design education. However, empirical evidence regarding their impact on students' critical thinking remains limited.

**Methods:**

To address this gap, this study employed a mixed-methods design involving a three-week intervention with 70 undergraduate design students. Participants were randomly assigned to an experimental group (*n* = 38) and a control group (*n* = 32), both with comparable academic backgrounds. Standardized instruments were used to measure students' critical thinking abilities before and after the intervention. During an age-friendly design workshop, the experimental group received AI-assisted feedback and assessment, while the control group completed the same instructional activities without AI support.

**Results:**

The results indicated that the experimental group demonstrated a statistically significant improvement in critical thinking abilities compared to the control group after the intervention (*p* < 0.05). Qualitative analysis further revealed several mechanisms through which AI-assisted feedback supported students' reflective thinking, iterative design processes, and problem-solving strategies.

**Discussion:**

These findings suggest that AI- effectively enhance critical thinking in design education. The study contributes empirical evidence to the growing body of research on AI-supported learning and provides practical implications for integrating AI tools into workshop-based design instruction to foster higher-order thinking skills.

## 1 Introduction

Critical thinking, as a crucial cognitive ability, emphasizes the independent and in-depth thinking that individuals undertake when analyzing information, evaluating arguments, and ultimately forming rational judgments and decisions (Dwyer, 2017; Krupat et al., 2011). It is not only regarded as the core goal of higher education, significantly promoting students‘ cognitive development and academic performance (Rivas et al., 2023, 2022), but also highly valued in today's labor market, becoming a key ability for graduates to achieve career success and actively participate in social life (Dumitru and Halpern, 2023). Therefore, exploring effective teaching methods to enhance students' critical thinking skills has become an urgent educational task.

In recent years, the rapid development of artificial intelligence technology has brought new dimensions and challenges to the cultivation of critical thinking. Artificial intelligence tools such as intelligent tutoring systems and adaptive learning platforms can create personalized and highly interactive learning environments, providing new possibilities for the development of higher-order thinking (Kassenkhan et al., 2025; Luo et al., 2025; Niu et al., 2025). Especially in the era of information explosion and technology-driven development, critical thinking is crucial for students to distinguish information and solve complex problems (Luo et al., 2024; Vu, 2025). How to effectively utilize artificial intelligence to overcome teaching bottlenecks, stimulate students' deep thinking and independent judgment, and achieve a good learning experience has become a core issue that must be addressed when combining technology with education (Balch and Loftus, 2023).

In the field of design education, the application of artificial intelligence is transforming the way students' creativity and analytical skills are cultivated in related professional courses. Some AI applications based on personalized feedback and scaffolded support are particularly important in design education, a field that highly relies on iteration and reflection. Tools driven by artificial intelligence, such as generative design systems and visualization platforms, enable students to quickly create prototypes and iterate designs, and receive contextual feedback throughout the design process, thereby forming an iterative and reflective learning cycle (Demartini et al., 2024; Serban and Vescan, 2019). These technologies can serve as powerful auxiliary tools to promote deeper cognitive engagement among students and stimulate creativity.

Taking this into account, the cultivation of critical thinking is particularly important in the design discipline, as design is essentially a decision-making process that balances creativity, functionality, ethics, and user needs (Verganti et al., 2021). An excellent designer needs to constantly evaluate the feasibility, appeal, and sustainability of their solutions, challenge existing assumptions, and integrate diverse perspectives. In the increasingly complex social-technical environment, the ability to critically assess the social, cultural, and environmental impacts of design has become indispensable. Therefore, emphasizing and cultivating critical thinking skills in design courses can help students cope with ambiguous situations in the future and enhance their innovative capabilities that are people-oriented and socially aware (Rusmann and Ejsing-Duun, 2022).

However, despite the increasingly widespread application of artificial intelligence in design education, existing research still has significant limitations. Most studies focus on the overall teaching effects of introducing AI tools, but generally lack comparative research on the impact of AI tools on students' critical thinking development. Current literature often lacks rigorous control groups in its experimental designs, making it difficult to accurately distinguish the extent to which improvements in critical thinking skills can be attributed to AI technology itself, rather than other teaching factors or students' natural development. This methodological gap limits our understanding of how AI promotes higher-order thinking development in design education and also affects the scientific optimization of related teaching strategies and tool designs.

This study focuses on whether artificial intelligence tools can enhance the critical thinking ability of design major students by providing personalized feedback and scaffolding learning experiences. The study adopts a mixed-methods approach to systematically compare the impact of using and not using AI tools on the development of students' critical thinking. A 3 week randomized controlled study was conducted. We recruited 70 undergraduate students from the design major of Fuzhou University in China. A *pre-test* was conducted to ensure that there were no significant differences between the experimental group and the control group in terms of baseline critical thinking levels, age, gender, and professional foundation. The experimental group integrated the use of ChatGPT-3.5 AI tools designed with prompts into their regular design courses, applying it to the analysis of design problems, the conception of solutions, and the evaluation of designs to provide personalized feedback and evaluation. The control group received the same number of class hours but traditional teaching without AI assistance, and both groups received unified feedback from the teacher at the end. Qualitative data such as learning process analysis and interviews were also collected to explore in depth the specific impact paths of AI tool intervention on critical thinking through a mixed-methods approach.

This study enriches the theoretical foundation of technology-enhanced learning in the field of design education by exploring the relationship between artificial intelligence and the cultivation of critical thinking in design majors. At the practical level, the findings of this study have direct guiding value for design educators, curriculum developers, and teaching administrators, providing evidence-based support for the reasonable integration of intelligent technology into the design teaching process. This helps educators create a more systematic and supportive learning environment in design courses, transforming artificial intelligence from an auxiliary tool into a teaching partner that promotes the development of deep design thinking.

## 2 Related work

### 2.1 Artificial intelligence in design education

Artificial intelligence technology has become a core driving force for educational innovation, providing a unique intelligent support for the teaching and learning process. In the field of design education, the application of artificial intelligence tools initially focused on knowledge-assisted skills training, such as how to better enhance cognitive abilities and the construction and acquisition of design knowledge (Zhang et al., 2026). In the field of design education, the application of artificial intelligence tools is gradually shifting from assisting skills training to supporting the cultivation of higher-order design thinking. In practice, artificial intelligence technologies such as generative design platforms and interactive web-based assessment tools can provide personalized support based on students' design process and outcomes, thereby meeting the diverse cognitive and creative needs of design learners. Lively et al. integrated AI-generated tools into web design education, using image- and text-based AI generators and software to enhance design students' aesthetic and creative copywriting skills (Lively et al., 2023).

In theory, Some researchers have also integrated certain concepts with artificial intelligence tools and design goals and principles. Roias et al. combined the 4PADAFE instructional design concept with artificial intelligence applications. 4PADAFE represents Academic Project, Strategic Plan, Instructional Planning, Instructional Material Production (4P), Teaching Action (AD), Formative Adjustments (AF), and Evaluation (E) to explore the possibilities and advantages of generative artificial intelligence tools in improving the teaching and learning process of design education (Ruiz-Rojas et al., 2023).

Existing research has begun to focus on the impact of artificial intelligence tools on the development of critical thinking, and related discussions are equally important in the context of design education. Some studies have shown that AI can promote students' reflection and argumentation in design decisions through structured process feedback, thereby fostering the development of critical thinking (Muthmainnah and Oteir, 2022; Zhao and Dang, 2026). This structured scaffolding adapts well to the timeliness of AI output, enabling students to experience multiple rounds of exploration and iteration in a short period, thus conducting in-depth thinking and critical analysis to enhance their design thinking patterns, creative thinking, and reflective thinking abilities (Saritepeci and Yildiz Durak, 2024). These cases demonstrate that artificial intelligence is not only a technological enhancement for design studies, but also a key means to propel design education to a deeper level and lay the foundation for students' design development.

### 2.2 Critical thinking in design

Critical thinking is often conceptualized as “the ability to think critically and make rational decisions in complex and uncertain situations.” This higher-order cognitive ability is rooted in Dewey's concept of “reflective thinking,” the core of which lies in the cyclical process of exploring, questioning, and evaluating design problems and solutions (Dewey, 1922; Rodgers, 2002).

In design, critical thinking is not merely a lofty cognitive concept, but a core skill deeply embedded in professional practice. The design process is essentially a cycle of continuous analysis, judgment, choice, and reflection, in which critical thinking naturally permeates every stage, from problem definition and solution generation to evaluation and optimization (Piotrowski, 2011).

Within design pedagogy, this competency becomes evident when learners rigorously dissect contextual design conditions, thoughtfully balance competing demands, such as functionality, sustainability, and user needs, critically assess not only their own proposals but also peer-generated alternatives, and substantiate design choices through logically structured, evidence-based reasoning (Tovey, 2015). Cognitively, critical thinking in design extends beyond evaluating pre-existing solutions; it actively fuels conceptual evolution by prompting designers to revisit, reinterpret, and sometimes fundamentally reframe their prior design experiences, thereby deepening domain-specific knowledge, refining conceptual frameworks, and strengthening metacognitive awareness (Findeli, 2001; Günther and Ehrlenspiel, 1999).

In design practice, critical thinking is often externalized and deepened through argumentation. Argumentation is defined here as a systematic process of presenting design propositions based on evidence and providing reasonable support for them (Günther and Ehrlenspiel, 1999). Designers not only need to generate ideas, but also need to be able to clearly articulate the basis, trade-offs, and logic behind design decisions. This is the specific manifestation of critical thinking in the design process. The elements such as “claim-evidence-basis-support-rebuttal” in the Toulmin argument model provide an effective framework for analyzing and constructing design arguments, helping students develop systematic and rational expression skills in design reviews, proposal defenses, and other aspects (Hitchcock, 2005; Toulmin, 2003).

To enhance students' critical thinking skills in design, structured argumentation has been widely adopted as an effective teaching intervention method. Although traditional approaches such as facilitating discussions and debates or structuring viewpoints have been proven to promote the development of critical thinking, they are often limited by issues like insufficient time and delayed feedback (Schindler and Burkholder Jr, 2014). In recent years, AI-driven argumentation tools have offered innovative solutions to this dilemma. However, while artificial intelligence-supported argumentation tools have shown significant potential, current research primarily focuses on the concepts and applications of the technology itself; how they differentially influence the critical thinking abilities of design students remains to be studied. Therefore, this study aims to construct a comparative experiment to explore the specific mechanisms by which AI-powered argumentation tools affect the development of students' critical thinking skills in design education, thereby filling a research gap in this field.

Therefore, this study aims to explore the impact of artificial intelligence tools on students' critical thinking abilities in designing instructional scenarios through a controlled experiment, and focuses on addressing the following two research questions:

Research Question 1: In university design courses, are there significant differences in critical thinking skills between students in the experimental group who used AI-assisted feedback and argumentation and those who used traditional design teaching methods?

Research Question 2: How did the critical thinking skills of the experimental group students change specifically in *pre-* and *post-test* assessments during the use of AI-assisted design argumentation tools?

## 3 Methodology

### 3.1 Participants

This study aimed to evaluate the impact of a 3 week workshop on “Age-Friendly Healthcare System Interface Design,” incorporating AI-assisted design tools, on the critical thinking skills of design students. A randomized controlled trial design was employed. This study adopted a randomized controlled trial design. Seventy undergraduate design students from a university in southeastern China were selected as the research subjects. After signing the informed consent form, they were randomly assigned to the experimental group (*n* = 38) and the control group (*n* = 32) in a 1:1 ratio using a computer-generated random sequence. Before the 4 week intervention began, we tested the baseline data of both groups to verify the effect of randomization. The results of independent sample *t*-tests and chi-square tests (Table 1) showed that there were no statistically significant differences between the two groups in key variables such as design foundation course grade points, gender, and design study duration (*p* > 0.05), indicating that the random grouping was effective and the groups had good comparability. After the intervention, all people in the experimental and control group completed the entire research process, and their data were included in the final analysis. To comply with academic ethics guidelines, this research was approved by the ethics review committee of the host university before it commenced. All participants signed informed consent forms, clearly explaining the research objectives, procedures, and their right to voluntarily withdraw. Considering potential copyright issues related to the design process and the work, all data during the research process was anonymized and kept strictly confidential.

**Table 1:** Participant demographics.

**Variable**	**Control group (*n* = 32)**	**Experimental group (*n* = 38)**	**Statistical test**	***p-*value**
**Age (years)**				
Mean ± SD	20.7 ± 1.3	20.9 ± 1.2	^*^t^*^= −0.68	0.501
**Gender**, ***n*** **(%)**				
Male	14 (43.8%)	18 (47.4%)	χ^2^ = 0.09	0.764
Female	18 (56.2%)	20 (52.6%)		
**Years of design study, n (%)**				
≤ 1 years	10 (31.2%)	14 (36.8%)	χ^2^ = 0.25	0.618
>1 years	22 (68.8%)	24 (63.2%)		
**GPA in foundation courses**				
Mean ± SD	3.51 ± 0.26	3.48 ± 0.28	^*^t^*^= 0.48	0.635

### 3.2 Experimental procedure

**Figure 1:**
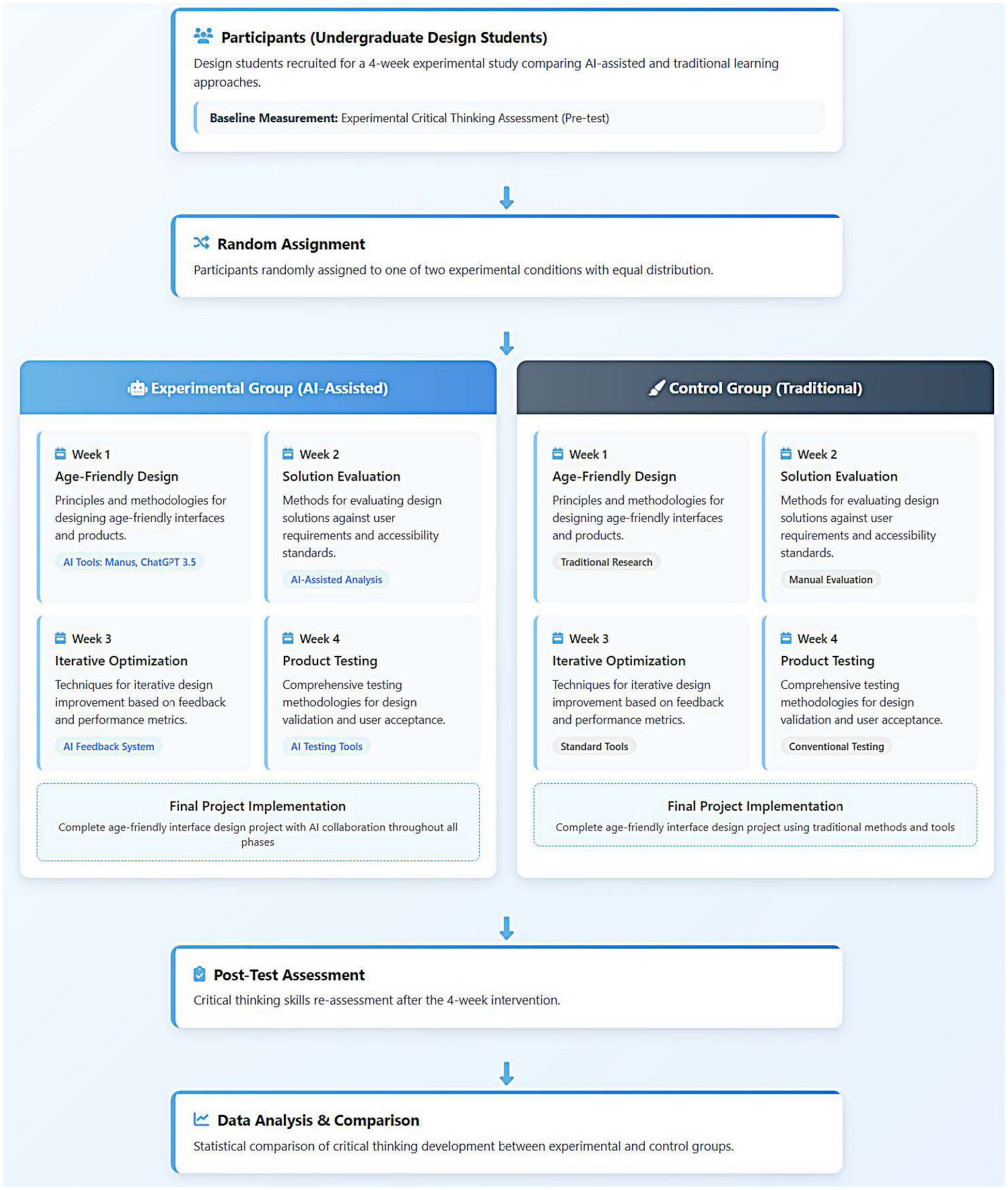
Workflow design process of the workshop experiment.

This study collected quantitative data through *pre-tests* and *post-tests* to assess the critical thinking abilities of design students. We conducted a 3 week “Age-Friendly Medical System Interface Design” workshop at Fuzhou University during the winter semester of 2025. The workshop was held once a week for 1 h, and was led by two researchers who had undergone unified training. Figure 1 shows the design of the experiment.

The experimental group received training on the following topics over the 4 weeks: “Age-Friendly Design,” “Solution Evaluation,” “Iterative Optimization,” and “Product Testing.” During the project implementation, students in this group were permitted and encouraged to use various applicable AI-assisted design tools, including Manus and Chatgpt 3.5. Students in the control group participated in the same workshop with the same theme, duration, and structure, but were prohibited from using any AI-based design tools during the project implementation; they were only allowed to use basic design software such as Sketch and Adobe to complete the tasks. The initial assessment involved students from both the experimental group and the control group. They completed *pre-tests* using relevant scales to determine their baseline levels of critical thinking. After the intervention, *post-tests* were conducted for both groups of students.

To obtain effective quantitative results, we first used paired tests to determine whether there were significant differences in *pre*- and *post*-*test* scores within each group, thus comparing the *pre-* and *post-test* results of the experimental and control groups. Paired *t*-tests were used when the data approximately followed a normal distribution; otherwise, Wilcoxon signed-rank tests were used.

To compare the effectiveness of the intervention, we used analysis of variance (ANOVA) to test whether there were significant differences in *post-test* scores between the experimental and control groups, controlling for *pre-test* results. We then combined the changes in *post-test* scores with those changes in pre-test scores to determine the final effect of the intervention.

In addition, this study also collected qualitative data through semi-structured interviews to gain a deeper understanding of the actual experiences and views of the students in the experimental group regarding the artificial intelligence-supported design tools. We conducted a 10 to 15 min interview with all the students in the experimental group, focusing on their experiences of using AI tools for design and exploration during the workshop. The content covered the impact of the tools on their design thinking, creative generation, solution optimization and other design learning processes, as well as the challenges they encountered regarding critical thinking in the human-machine collaborative design process.

**Figure 2:**
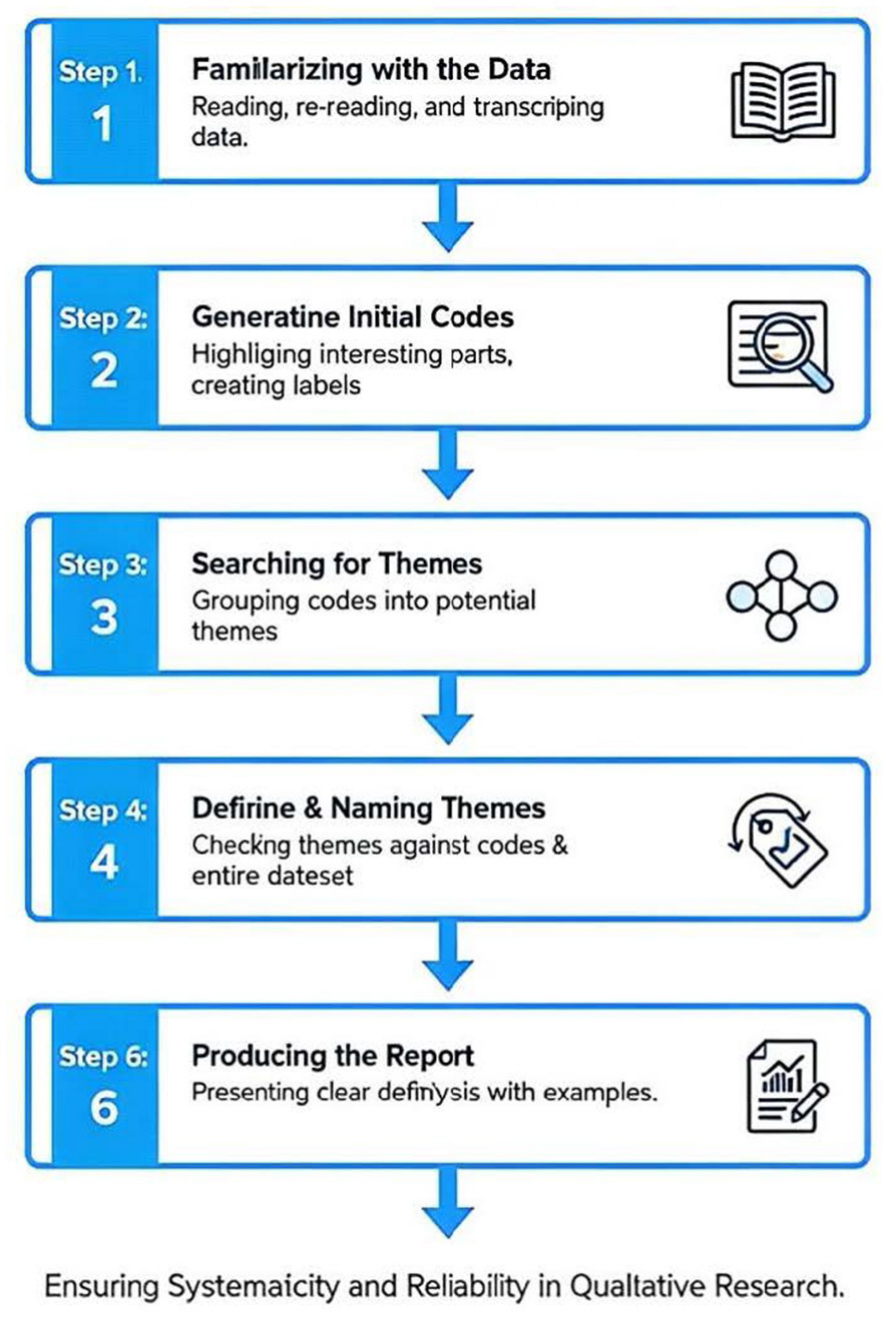
Thematic analysis process.

The interview outline was mainly composed of open-ended questions to encourage participants to fully express their thoughts and insights. Subsequently, this study used Maxqda software to conduct a thematic analysis of the interview texts, aiming to identify common themes related to critical thinking in design learning in the students' responses. The thematic analysis strictly followed the six-step framework proposed by Braun and Clarke to ensure the systematicity and reliability of the analysis process, whic as shown in Figure 2 (Braun and Clarke, 2006): (1) Familiarizing with the data: reading the interview transcripts to form an initial understanding; (2) Generating initial codes: marking the content units of the text; (3) Searching for themes: clustering related codes to form the initial shape of potential themes; (4) Reviewing themes: checking the matching degree between themes and codes, and optimizing the theme structure; (5) Defining and naming themes: precisely defining the connotation of each theme and naming it; (6) Writing the report: selecting representative original materials to clearly present the analysis results in the research. This analysis helps to understand the impact of AI tools on students' critical thinking in their professional learning experience from a design perspective, thereby effectively complementing the quantitative research results.

This study had already undergone review and approval by the ethics committee of the host university before its implementation. All participants were informed of the purpose of the study, the process and potential risks, and signed an informed consent form before data collection. Participation was entirely voluntary, and students were informed that they had the right to withdraw from the study at any time without facing any penalties. To protect the privacy and confidentiality of the participants, all collected data have been anonymized. Additionally, any design works and learning records generated during the seminar are regarded as confidential research materials and will not be used for any non-research purposes or evaluations.

### 3.3 Instruments

#### 3.3.1 Critical thinking assessment tool

This study adapted the Chinese version of the Critical Thinking Disposition Scale (CTDI-CV) to assess students' critical thinking levels before and after intervention. The original scale assesses seven aspects of critical thinking: truth-seeking, openness, analytical thinking, systematic thinking, confidence in critical thinking, curiosity, and cognitive maturity, using a 6-point Likert scale, ranging from 1 (strongly disagree) to 6 (strongly agree). Higher scores indicate a stronger tendency toward critical thinking. While the original scale comprises 70 items across seven dimensions, a shortened version was developed to minimize participant fatigue and ensure response quality within the intensive design workshop context. Through a systematic content validity evaluation, two design education experts and one psychometrician reviewed the items to ensure alignment with the design-thinking process. Ultimately, it retains five dimensions suitable for design research: openness, analytical thinking, systematic thinking, confidence in critical thinking, and cognitive maturity. These dimensions are all closely related to design. Items were selected based on their factor loadings in previous validation studies and their relevance to creative problem-solving. Finally, three questions were chosen for each dimension to ensure their linguistic and cultural context was consistent with the design context.

To ensure the validity of the scale, we measured its reliability and validity. The overall content validity index (CVI) of the CTDI-CV is 0.89, indicating satisfactory content validity. To evaluate the internal consistency of the scale, a reliability analysis was conducted. The overall Cronbach's α coefficient of the CTDI-CV is 0.90, and the α coefficients of each subscale range from 0.61 to 0.74, reflecting satisfactory internal consistency for each dimension.

## 4 Results

### 4.1 Quantitative analysis results

This section presents the quantitative and qualitative analysis results of the CTDI-CV scores. Table 2 summarizes the descriptive statistics of the *pre-test* and *post-test* scores of the experimental and control groups. It shows that the average score of the experimental group increased by 3.29 points, while that of the control group only increased by 0.81 points. This indicates that the progress of the experimental group was more significant. The experimental group also demonstrated greater dispersion in the differences between the pre-test and post-test, with a wider range of individual variations, suggesting that the intervention effect may be more significant among different individuals.

**Table 2:** Descriptive statistics for pre-test and post-test scores by group.

**Group**	***n***	**Pre-test mean ±SD**	**Post-test mean ±SD**	**Mean difference ±SD**	**Range of difference**
Control	32	51.31 ± 6.19	52.12 ± 6.38	0.81 ± 3.15	[−7, 7]
Experimental	38	52.08 ± 7.02	55.37 ± 7.14	3.29 ± 3.76	[−1, 16]

Table 3 presents the results of paired comparisons between *pre-test* and *post-test*. After conducting a normality test, it was found that the differences between the pre- and post-tests for both groups did not satisfy the assumption of normal distribution (Shapiro–Wilk test, *p* < 0.05). Therefore, the Wilcoxon signed-rank test (Wilcoxon symbol rank test) was used to examine the differences in the pre- and post-test scores between the experimental group and the control group.

**Table 3:** Paired comparisons between pre-test and post-test scores.

**Group**	**Normality test (Shapiro–Wilk)**	**Statistical test**	***W***	***p*-value**	**Conclusion**
Control	*W* = 0.9013, *p* = 0.0067	Wilcoxon signed-rank test	77	0.1039	Not significant
Experimental	*W* = 0.8667, *p* = 0.0003	Wilcoxon signed-rank test	7	<0.001	Significant (Post-test > Pre-test)

The results showed that although the control group exhibited a certain degree of score improvement between the pre-test and post-test, this difference did not reach a statistically significant level (*p* = 0.1039). This indicates that under the condition of no intervention, the variation in students' scores is limited, and it may mainly reflect the time effect. In contrast, the experimental group showed a larger improvement between the pre-test and post-test, and the difference reached a statistically significant level (*p* < 0.001), indicating that under the experimental condition, students' performance improved significantly.

It should be noted that the paired test analysis only compares the pre-test and post-test within the same group. It cannot directly draw a causal conclusion that the experimental group is superior to the control group. The significant improvement in the experimental group may be related to the intervention, but it could also be influenced by factors such as initial level differences, individual differences, and other factors (Niu et al., 2025). Therefore, further tests using inter-group comparison methods are still needed to verify whether the difference in post-test performance between the experimental group and the control group is significant after controlling for the pre-test levels.

To test whether there were differences in the post-test scores between the experimental group and the control group after controlling for the pre-test scores, we conducted an ANOVA. In this model, the post-test scores were the dependent variable, the group (experimental group vs. control group) was the fixed factor, and the pre-test scores were the covariate. Table 3 presents the results of the ANOVA unstandardized regression coefficients. As shown in Table 4, the covariate (pre-test scores) could significantly predict the post-test scores (*p* < 0.001), indicating a significant correlation between the baseline scores and the outcome scores. Importantly, after adjusting for the pre-test scores, the group effect remained statistically significant (*B* = 2.57, *p* = 0.003), suggesting that the post-test scores of the experimental group were significantly higher than those of the control group.

**Table 4:** ANOVA regression results.

**Predictor**	***B***	***SE***	***t***	***p*-value**	**95% CI**	**Partial Eta Squared**
Intercept	6.7547	3.267	2.068	0.043	[0.234, 13.276]	
Group (Experimental vs. Control)	2.5657	0.825	3.108	0.003	[0.918, 4.213]	0.126
Pre-test score	0.8842	0.063	14.133	<0.001	[0.759, 1.009]	0.749

The observed net gain of 2.57 points carries substantial pedagogical weight. In terms of effect size, the Partial Eta Squared indicates a medium-to-large effect size, suggesting that the intervention independently explains 12.6% of the variance in learning outcomes after adjusting for initial proficiency. This demonstrates that the teaching model is not only statistically significant but also has strong applicability in actual teaching practice. This difference represents approximately 0.40 standard deviations. In an educational context, this magnitude of improvement suggests that an average student receiving the intervention would outperform 66% of students in the control group. This result indicates that the intervention received by the experimental group did not merely produce a natural improvement related to the time effect, but rather generated an additional promoting effect beyond that of the control condition.

Based on the comprehensive analysis results within and between groups, although the control group did not show significant improvement, the experimental group not only achieved significant progress in its own pre- and post-tests, but also remained significantly superior to the control group after controlling for baseline differences. This to some extent supports the conclusion that feedback and evaluations from artificial intelligence tools have a positive effect on enhancing the critical thinking skills of design students.

### 4.2 Qualitative analysis results

This thematic analysis focused on exploring how feedback and evaluation activities supported by artificial intelligence can promote the development of critical thinking among design students. From the interview data, three interrelated themes were extracted: (1) Cognitive participatory evaluation activities; (2) Feedback-driven reflective activities; (3) Scaffolded analytical reasoning.

Firstly, assessment activities supported by artificial intelligence are believed to facilitate higher levels of cognitive engagement. For instance, Student 4 stated that artificial intelligence-assisted debates encourage them to “express their ideas more clearly,” and the immediate feedback during the debate process helps them re-evaluate and refine their arguments (Student 3 and 7). Some students repeatedly emphasized in the interviews the importance of the immediate and personalized feedback provided by artificial intelligence tools, which enables them to identify errors, recognize misunderstandings, and reflect on their reasoning processes (Student 12 and 22).

Furthermore, some students emphasized that the AI-assisted design tools helped them organize their thoughts, making the relationships between concepts clearer, thereby facilitating deeper connections among design elements. For instance, Student 10 said: “I thought that elderly-friendly design should have larger fonts in the application, but I forgot that some pop-up windows also require a longer time.” Additionally, the text-related questions generated by AI encouraged students to go beyond the comprehension level and explain, analyze, and evaluate the learning materials (Students 13 and 15).

## 5 Discussion

This study investigated the impact of artificial intelligence-assisted tools on the development of critical thinking skills among design students. Both quantitative and qualitative analysis results indicated that the feedback and assessment practices facilitated by artificial intelligence could effectively enhance students' higher-order thinking abilities and positively contribute to the development of critical thinking. The thematic analysis revealed that students generally believe that learning activities supported by artificial intelligence can significantly enhance their participation in design, and encourage them to more actively express and defend their own viewpoints during the learning process, while considering different perspectives and possible solutions. For design disciplines centered on creative problem-solving, this is particularly important, as the design process itself heavily relies on the analytical and evaluative dimensions of critical thinking (Shively et al., 2018).

This result is also consistent with many previous studies. Arseven and Bal research demonstrates that when students use artificial intelligence-assisted tools, they do not merely rely on simple copying and pasting; instead, they assist in thinking training (Arseven and Bal, 2025). This also reveals that artificial intelligence is not merely a simple tool but serves as a mechanism to construct various elements of design activities and guide design learning students in analyzing, evaluating, and reflecting on the alignment between design goals and their own thoughts.

Furthermore, students repeatedly mentioned that the immediate feedback and correction suggestions provided by artificial intelligence help to form the iterative learning cycle of “feedback-reflection-revision” in critical thinking (Atkinson et al., 2008; Luo et al., 2025). Existing research generally holds that this continuous feedback and correction process is a key mechanism for reflective learning, and it also the important foundation for the development of critical thinking. In terms of design education, this learning method is not merely about mastering knowledge, but rather a cognitive and creative process that integrates practical orientation and humanistic care, which helps students constantly examine, correct, and improve their design judgments in real problem situations (Buchanan, 2004). For example, in the case study of age-friendly design in this research, this field not only involves functionality and usability issues, but also relies heavily on the analysis and evaluation of the usage context, ethics, and humanistic considerations. These are precisely the keys to the success of the design (Liu et al., 2026, 2025).

In the workshop teaching scenario of this study, although the control group showed a slight improvement between the pre-test and post-test, this change did not reach the statistical significance level. This indicates that relying solely on the passage of time or routine classroom activities to generate a time effect is insufficient to significantly promote students‘ critical thinking development in the short term. This result to some extent reflects that, within the limited classroom time, teachers find it difficult to attend to all students and provide targeted feedback support continuously. For design disciplines that focus on critical judgment and reflective decision-making, this problem may be particularly prominent. The findings of this study also indirectly suggest that in short-term workshop teaching, simply “learning by doing” may not be sufficient to promote significant development of critical thinking, and a timely feedback mechanism is needed to more effectively facilitate students' transition from experiential activities to deep cognitive processing.

## 6 Implication and limitation

Based on these findings, this study provides several practical implications for integrating workshop-based teaching and artificial intelligence tools in design education. Firstly, the research results indicate that merely relying on practical activities and time accumulation in workshops may not effectively promote the development of students' critical thinking in the short term. Therefore, in design tasks that are based on user-centered design and are suitable for the elderly, it is necessary to introduce more structured cognitive support mechanisms to compensate for the practical limitations of teachers, who may have difficulty providing continuous and personalized guidance to all students within the limited teaching time.

Secondly, the value demonstrated by the artificial intelligence tools in this study does not lie in replacing teachers. Instead, it lies in serving as a cognitive framework that can be used for self-thinking training and evaluation. By embedding these artificial intelligence tools into some key aspects of the design workshops, it can effectively enhance students' reflective and self-regulated learning processes, thereby promoting their critical thinking development at the analytical and evaluative levels.

Furthermore, this study has some limitations. The main limitation is that self-report measurement relying on critical thinking may introduce bias. This is particularly true for care-oriented design, where practical outputs may be more relevant than self-report methods. Additionally, the sample size of this study, limited to only one university in China, may restrict the general applicability of the results. Future research should include larger and more diverse samples and employ multiple data collection methods based on behavior or practice to validate the findings. Furthermore, due to discrepancies between the measurement dimensions and the theoretical framework. The CTDI-CV scale used in this study measures students‘ critical thinking inclination during the design process—that is, their willingness to actively reflect and analyze—but this still differs from their actual critical thinking skills. Although data demonstrates that AI tools significantly enhance students' thinking inclination, this does not directly equate to a substantial improvement in their argumentation, evaluation, and decision-making abilities. Future research should introduce process-based task assessments to further verify whether improvements in thinking inclination can effectively translate into higher-quality actual design outputs.

## 7 Conclusion

This study explored the role of artificial intelligence-assisted tools in supporting the development of critical thinking among design students. Quantitative results indicated that, after controlling the pre-test level, the experimental group that used the artificial intelligence tools performed significantly better in the post-test in terms of critical thinking compared to the control group that did not use the tool. This result suggests that the learning intervention provided by the artificial intelligence-assisted tools can effectively enhance students' critical thinking in design. Qualitative analysis further revealed the possible mechanisms behind this effect, including promoting students' reflection and revision during the design process, which supports their cognitive development at the level of analysis and evaluation. Our experiment also demonstrated the positive effect of introducing artificial intelligence-assisted design tools on the development of students' advanced cognitive abilities. We recommend establishing structured courses in design education to better utilize the feedback and evaluation functions of artificial intelligence. This study not only contributes to the application of artificial intelligence in design education but also provides practical verification of the effectiveness of artificial intelligence technology.

## Data Availability

The raw data supporting the conclusions of this article will be made available by the authors, without undue reservation.

## References

[B1] Arseven T. Bal M. (2025). Critical literacy in artificial intelligence assisted writing instruction: a systematic review. Think Skills Creat. 57:101850. doi: 10.1016/j.tsc.2025.101850

[B2] Atkinson L. C. O‘hair M. J. O'hair H. D. Williams L. A. (2008). Developing and sustaining schools as technology-enriched learning organizations. i-Manag. J. Sch. Educ. Technol. 3:17. doi: 10.26634/jsch.3.4.644

[B3] Balch J. A. Loftus T. J. (2023). Actionable artificial intelligence: overcoming barriers to adoption of prediction tools. Surgery 174, 730–732. doi: 10.1016/j.surg.2023.03.019 37198040 PMC12288832

[B4] Braun V. Clarke V. (2006). Using thematic analysis in psychology. Qual. Res. Psychol. 3, 77–101. doi: 10.1191/1478088706qp063oa

[B5] Buchanan R. (2004). Human-centered design: Changing perspectives on design education in the East and West. Des Issues 20, 30–39. doi: 10.1162/074793604772933748

[B6] Demartini C. G. Sciascia L. Bosso A. Manuri F. (2024). Artificial intelligence bringing improvements to adaptive learning in education: a case study. Sustainability 16:1347. doi: 10.3390/su16031347

[B7] Dewey J. (1922). An analysis of reflective thought. J. Philos. 47, 29–38. doi: 10.2307/2939444

[B8] Dumitru D. Halpern D. F. (2023). Critical thinking: creating job-proof skills for the future of work. J. Intell. 11:194. doi: 10.3390/jintelligence11100194 37888426 PMC10607682

[B9] Dwyer C. P. (2017). Critical Thinking. Cambridge: Cambridge University Press. doi: 10.1017/9781316537411

[B10] Findeli A. (2001). Rethinking design education for the 21st century: theoretical, methodological, and ethical discussion. Des. Issues 17, 5–17. doi: 10.1162/07479360152103796

[B11] Günther J. Ehrlenspiel K. (1999). Comparing designers from practice and designers with systematic design education. Des. Stud. 20, 439–451. doi: 10.1016/S0142-694X(99)00019-8

[B12] Hitchcock D. (2005). Good reasoning on the Toulmin model. Argumentation 19, 373–391. doi: 10.1007/s10503-005-4422-y

[B13] Kassenkhan A. M. Moldagulova A. N. Serbin V. V. (2025). Gamification and Artificial Intelligence in Education: A Review of Innovative Approaches to Fostering Critical Thinking. New York, NY: IEEE Access. doi: 10.1109/ACCESS.2025.3576147

[B14] Krupat E. Sprague J. M. Wolpaw D. Haidet P. Hatem D. O'Brien B. (2011). Thinking critically about critical thinking: ability, disposition or both? Med. Educ. 45, 625–635. doi: 10.1111/j.1365-2923.2010.03910.x 21564200

[B15] Liu T. Luo Y. T. Pang P. (2026). How Older Adults Use Digital Technologies for Healthcare? A systematic scoping review. J. Med. Syst. 50:5. doi: 10.1007/s10916-025-02331-8 41498996

[B16] Liu T. Luo Y. T. Pang P. C.-I. (2025). Digital technologies-enhanced older adults health management: developing a five-dimensional extension of social learning theory for community settings. Front. Public Health 13:1627983. doi: 10.3389/fpubh.2025.1627983 40761933 PMC12318761

[B17] Lively J. Hutson J. Melick E. (2023). Integrating AI-Generative tools in web design education: enhancing student aesthetic and creative copy capabilities using image and text-based AI generators. J. Artificial Intelligence Robot. 1, 23-26. doi: 10.59232/AIR-V1I1P103

[B18] Luo Y. Pang P. C.-I. Chang S. (2024). “Enhancing exploratory learning through exploratory search with the emergence of large language models,” in Proceedings of the 58th Hawaii International Conference on System Sciences (HICSS 2025), 44–53. doi: 10.24251/HICSS.2025.007

[B19] Luo Y. T. Liu T. Pang P. C.-I. Wang Z. Chan K. I. (2025). Exploring information interaction preferences in an llm-assisted learning environment with a topic modeling framework. Appl. Sci. 15:7515. doi: 10.3390/app15137515

[B20] Muthmainnah Ibna Seraj, P. M. Oteir I. (2022). Playing with AI to investigate human-computer interaction technology and improving critical thinking skills to pursue 21st century age. Educ. Res. Int. 2022:6468995. doi: 10.1155/2022/6468995

[B21] Niu T. Liu T. Luo Y. T. Pang P. C.-I. Huang S. Xiang A. (2025). Decoding student cognitive abilities: a comparative study of explainable AI algorithms in educational data mining. Sci. Rep. 15:26862. doi: 10.1038/s41598-025-12514-5 40702127 PMC12287387

[B22] Piotrowski C. M. (2011). Problem Solving and Critical Thinking for Designers. Hoboken, NJ: John Wiley and Sons.

[B23] Rivas S. F. Saiz C. Almeida L. S. (2023). The role of critical thinking in predicting and improving academic performance. Sustainability 15:1527. doi: 10.3390/su15021527

[B24] Rivas S. F. Saiz C. Ossa C. (2022). Metacognitive strategies and development of critical thinking in higher education. Front. Psychol. 13:913219. doi: 10.3389/fpsyg.2022.913219 35783800 PMC9242397

[B25] Rodgers C. (2002). Defining reflection: another look at John Dewey and reflective thinking. Teach. College Rec. 104, 842–866. doi: 10.1111/1467-9620.00181

[B26] Ruiz-Rojas L. I. Acosta-Vargas P. De-Moreta-Llovet J. Gonzalez-Rodriguez M. (2023). Empowering education with generative artificial intelligence tools: Approach with an instructional design matrix. Sustainability 15, 11524. doi: 10.3390/su151511524

[B27] Rusmann A. Ejsing-Duun S. (2022). When design thinking goes to school: a literature review of design competences for the K-12 level. Int. J. Technol. Design Educ. 32, 2063–2091. doi: 10.1007/s10798-021-09692-4

[B28] Saritepeci M. Yildiz Durak H. (2024). Effectiveness of artificial intelligence integration in design-based learning on design thinking mindset, creative and reflective thinking skills: an experimental study. Educ. Inf. Technol. 29, 25175–25209. doi: 10.1007/s10639-024-12829-2

[B29] Schindler L. Burkholder Jr G. J. (2014). Instructional design and facilitation approaches that promote critical thinking in asynchronous online discussions: a review of the Literature. High. Learn. Res. Commun. 4, 24-41. doi: 10.18870/hlrc.v4i4.222

[B30] Serban C. Vescan A. (2019). “Advances in designing a student-centered learning process using cutting-edge methods, tools, and artificial intelligence: an e-learning platform,” in Proceedings of the 1st ACM SIGSOFT International Workshop on Education Through Advanced Software Engineering and Artificial Intelligence. doi: 10.1145/3340435.3342716

[B31] Shively K. Stith K. M. Rubenstein L. D. (2018). Measuring what matters: assessing creativity, critical thinking, and the design process. Gifted Child. Today 41, 149–158. doi: 10.1177/1076217518768361

[B32] Toulmin S. E. (2003). The Uses of Argument. Cambridge: Cambridge university press. doi: 10.1017/CBO9780511840005

[B33] Tovey M. M. (2015). Design Pedagogy: Developments in Art and Design Education. Surrey: Ashgate Publishing, Ltd.

[B34] Verganti R. Dell'Era C. Swan K. S. (2021). Design thinking: critical analysis and future evolution. J. Product Innov. Manag. 38, 603-622. doi: 10.1111/jpim.12610

[B35] Vu H. X. (2025). Leveraging technology for higher-order thinking development: Instructional design strategies in higher education. Int. J. Technol. Learn. 32:25. doi: 10.18848/2327-0144/CGP/v32i02/25-46

[B36] Zhang P. Rai J. S. Almugren I. Pironti M. Derhy A. (2026). Generative AI adoption in higher education. Knowledge management perspective on application, acquisition and entrepreneurial skill development. J. Knowled. Manag. 30, 1-25. doi: 10.1108/JKM-10-2025-1426

[B37] Zhao H. Dang T. N. Y. (2026). Transforming written assessment design to embrace AI: what needs to be changed to encourage higher-order critical thinking. Educ. Inform. Technol. 31, 1-23. doi: 10.1007/s10639-025-13870-5

